# High Output Heart Failure Following Leadless Pacemaker Implantation

**DOI:** 10.1016/j.jaccas.2026.107012

**Published:** 2026-03-11

**Authors:** Tawfiq A. Khasawneh, Lyle H. Miller, Noah J. Blaker, Ramil K. Goel, Robert F. Hamburger

**Affiliations:** a Division of Cardiovascular Medicine, University of Florida, Gainesville, Florida, USA; b Malcom Randall VA Medical Center, Gainesville, Florida, USA

**Keywords:** acute heart failure, cardiac pacemaker, vascular disease

## Abstract

**Background:**

Leadless pacemakers have a significantly higher risk of vascular complications than traditional transvenous devices.

**Case Summary:**

An 85-year-old man with permanent atrial fibrillation presented with symptomatic bradycardia. A leadless pacemaker was implanted, and the procedure was complicated by a right common femoral vein pseudoaneurysm which was treated with thrombin injection. Months later, he presented with worsening volume overload related to high output heart failure from an arteriovenous fistula at the access site. After surgical repair of his fistula, the patient had resolution of heart failure.

**Discussion:**

Arteriovenous fistula is a relatively rare complication of cardiac catheterization, but its risk is increased with procedures like leadless pacemaker insertion. Such procedures require a larger caliber sheath, which is associated with a significantly higher risk of vascular complications.

**Take-Home Messages:**

Leadless pacemakers have a unique set of associated vascular complications that could lead to significant adverse outcomes.

## Graphical Abstract

**Figure:**
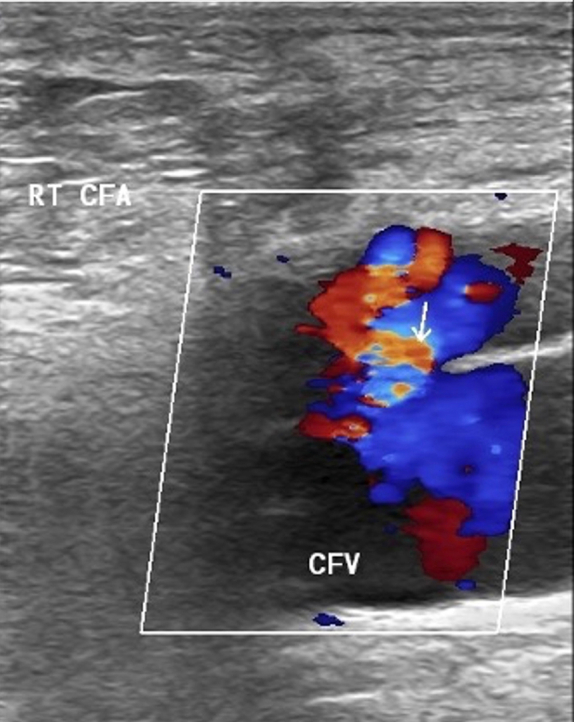

## History of Presentation

An 85-year-old man with a prior history of permanent atrial fibrillation and symptomatic bradycardia status post leadless pacemaker implant a few months before admission presented to our facility with complaints of shortness of breath, bilateral lower extremity swelling, and a 14 kilogram weight gain. On physical examination, he was found to have jugular venous distension, anasarca, and a palpable thrill and an audible bruit over his prior right common femoral venous access site. His admission N-terminal pro–B-type natriuretic peptide was 14,542 pg/mL.

## Past Medical History

The patient had a history of permanent atrial fibrillation with slow ventricular response. He had been hospitalized several months prior at an outside hospital due to symptomatic bradycardia requiring placement of a permanent pacemaker. The patient underwent landmark-guided implantation of a leadless pacemaker; however, the procedure was complicated by pseudoaneurysm formation requiring interventional radiology-guided thrombin injection. During that admission, a transthoracic echocardiogram (TTE) showed a left ventricular ejection fraction (LVEF) of 60% to 65% and normal diastolic function. Follow-up ultrasounds immediately and 6 weeks post-thrombin injection showed no residual flow within the pseudoaneurysm, with the latter showing fluid collections at the level of the common femoral artery and vein.

## Differential Diagnosis

Given the symptoms of paroxysmal nocturnal dyspnea, orthopnea, worsening edema, and significantly elevated N-terminal pro–B-type natriuretic peptide, acute heart failure was the leading diagnosis. Other less likely considerations included cirrhosis, symptomatic pulmonary hypertension, and nephrotic syndrome; however, these were quickly excluded.

## Investigations

**Figure 1:**
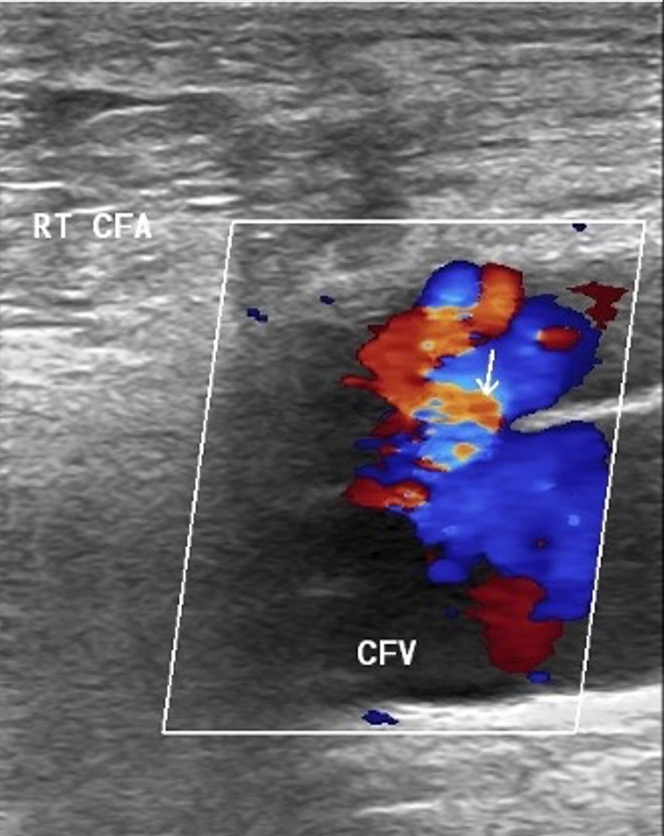
Arteriovenous Fistula Between the RT CFA and CFV Arrow indicates arteriovenous fistula. CFA = common femoral artery; CFV = common femoral vein; RT = right.

Duplex ultrasound was performed to further evaluate his palpable thrill and revealed a significant right femoral artery to common femoral vein fistula (Figure 1). TTE was then performed showing preserved LVEF of 60% to 65%, normal diastolic function, and mild right ventricular dilation.

## Management (Medical/Interventions)

The patient received aggressive intravenous diuresis with furosemide with improvement in volume status and was discharged on oral furosemide. He was referred to vascular surgery and underwent repair of the AV fistula with a bovine pericardial patch.

## Outcome and Follow-Up

On subsequent clinic follow-up, after surgical repair, he was found to be asymptomatic and euvolemic off all diuretics.

## Discussion

Leadless pacemakers have several advantages in comparison with traditional transvenous pacemakers. These advantages are related to the lack of leads, generator pockets, and increased patient comfort.^1^^,^^2^ Lack of leads and generator pockets allow for significantly lowered risk of infections from 0.7% to <0.2% (*P* < 0.0001), as demonstrated by Crossley et al.^1^ The same study showed a 32% lower rate of chronic complications, and a 41% lower rate of reinterventions compared with the traditional transvenous pacemaker.^1^ In addition to the lowered risk of infection and chronic complications, the device is also appealing due to increased patient comfort due to absence of a generator pocket, with thus no postoperative pain related to the pocket site, less movement restrictions immediately after the operation, and improvement in overall quality of life.^2^

Although leadless pacemakers have many benefits, they also have several drawbacks. Multiple studies have identified that although the overall rate of complications is lower over the medium term, they have also found that the complications that do occur are predominantly vascular in nature with a significantly increased risk of hematomas, pseudoaneurysm, and fistulas.^1^^,^^3^ Alhuarrat et al^4^ found that the risk for vascular complications is >7 times higher in patients receiving a leadless device vs a traditional transvenous device (OR: 7.54; *P* < 0.001). This significantly increased risk is likely due to the 27-F catheter (9-mm diameter) combined with the increased severity of comorbidities in the population that generally receives these devices.^4^ The requirement of femoral venous access for leadless pacemakers, compared with axillary/subclavian venous access for traditional pacemaker/implantable cardioverter-defibrillator leads also likely increased the risk of vascular complications due to similar factors—greater depth of femoral vessels and patient mobility affecting proper access site hemostasis.

Iatrogenic pseudoaneurysms post cardiac catheterizations are uncommon but easily managed with thrombin injections, with success rates surpassing 90%.^5^^,^^6^ Imaging follow-up within 24 hours and at 3 to 4 weeks postinjection is generally used to evaluate for complications and success.^5^^,^^7^

Arteriovenous fistula (AVF) is a generally rare occurrence after diagnostic and therapeutic cardiac catheterization, which occurs due to an unrecognized combined arterial and venous puncture.^8^ In a study aiming to measure the incidence of AVF in femoral access, <1% of patients experienced an AVF. Around one-third of arteriovenous communications closed spontaneously by 1 year, and most that persisted remained asymptomatic.^8^ These figures are, however, not representative of leadless pacemaker implantation because the larger sheath size could contribute to an increased risk of more severe and frequent complications, as resulting AV communications have the potential to be larger than in other cardiac procedures. This could ultimately lead to a lower rate of spontaneous closure of AVF, and more symptomatic presentations, as the large flow through the fistula could lead to distal claudication or in the case of our patient, high output heart failure.

The increased risk of complications makes it paramount to adopt risk-reducing strategies during such procedures. In a meta-analysis comparing ultrasound guidance vs landmark guidance for vascular access in electrophysiological procedures, Triantafyllou et al^9^ found that ultrasound guidance led to a near 60% reduction in vascular complications (relative risk: 0.38; 95% CI: 0.27-0.53). The substantial improvement in outcomes associated with use of ultrasound guidance made it standard of care in many institutions.

In this case, the patient began to develop clinical signs of heart failure several weeks after leadless pacemaker placement complicated by iatrogenic pseudoaneurysm requiring thrombin injection. This occurred despite a normal echocardiogram at the time of pacemaker implantation, no prior history of heart failure, and appropriate follow-up ensuring resolution of pseudoaneurysm. Imaging at the time revealed an AVF that was surgically repaired with subsequent resolution of his heart failure, which confirms the diagnosis of high-output heart failure secondary to an iatrogenic AVF after his pacemaker placement. The uniqueness of our case lies in the large communication between the femoral artery and vein resulting in a magnitude of AV shunting resulting in clinical high output heart failure. A review of the original operative report for the implantation of the pacemaker revealed that femoral venous access was obtained without ultrasound guidance. We think the use of ultrasound guidance could have potentially avoided this complication and illustrates the importance of using the imaging tool, especially when obtaining access for large bore sheaths.

## Conclusions

Although leadless pacemakers have an advantage over traditional pacemakers in device-related complications, especially in the setting of infection, they present a significant risk of vascular complications that should not be overlooked.

## Funding Support and Author Disclosures

The authors have reported that they have no relationships relevant to the contents of this paper to disclose.

**Take-Home Messages**

• Leadless pacemakers have a unique set of associated vascular complications which should be considered when selecting a device for implantation.
• Any AV communication resulting from these procedures can be sizable and lead to clinically significant adverse outcomes.
• Ultrasound-guided vascular access can potentially decrease the incidence of these complications.

**Visual Summary:** Timeline of the Case

Date	Event
7 mo prior	85-year-old man with permanent atrial fibrillation presented with symptomatic bradycardia. A leadless pacemaker was implanted. Echocardiogram at the time showed LVEF of 60%-65% with no diastolic dysfunction.
1 wk after procedure	Patient experienced persistent discomfort and pain at the access site in the right groin. Ultrasound revealed a serpentine pseudoaneurysm of the right femoral artery. Patient subsequently underwent IR-guided thrombin ablation of the pseudoaneurysm.
6 wk after procedure	Ultrasound confirmed no residual flow with the pseudoaneurysm.
Hospital day 1	Patient presented to the ED with complaints of shortness of breath, bilateral lower extremity swelling, and a 30-lb weight gain. On physical examination, he was found to have jugular venous distension, anasarca, and a palpable thrill and an audible bruit over his prior right common femoral venous access site. His admission NT-proBNP was 14,542 pg/mL. Aggressive diuresis with intravenous furosemide was initiated.
Hospital day 2	Duplex ultrasound revealed a significant right femoral artery to common femoral vein fistula. TTE was then performed showing preserved LVEF of 60%-65%, normal diastolic function, and mild right ventricular dilation.
Hospital day 3-8	The diagnoses of high output heart failure secondary to femoral arteriovenous fistula was made. Patient was continuously diuresed and later transitioned to oral furosemide. Patient was discharged on hospital day 8 with a referral to vascular surgery for repair.
1.5 mo after admission	Patient underwent successful AVF repair with a bovine pericardial patch.
2 mo post repair	Patient was seen in clinic for follow-up and was found to be asymptomatic and off all diuretics.

AVF = arteriovenous fistula; ED = emergency department; IR = interventional radiology; LVEF = left ventricular ejection fraction; NT-proBNP = N-terminal pro–B-type natriuretic peptide; TTE = transthoracic echocardiogram.
